# Preoperative prediction of microvascular invasion of hepatocellular carcinoma with IVIM diffusion-weighted MR imaging and Gd-EOB-DTPA-enhanced MR imaging

**DOI:** 10.1371/journal.pone.0197488

**Published:** 2018-05-17

**Authors:** Wei Zhao, Wenguang Liu, Huaping Liu, Xiaoping Yi, Jiale Hou, Yigang Pei, Hui Liu, Deyun Feng, Liyu Liu, Wenzheng Li

**Affiliations:** 1 Department of Radiology, Xiangya Hospital of Centre-south University, Changsha, Hunan, P.R. China; 2 Department of Radiology, Huadong Hospital Affiliated to Fudan University, Shanghai, P.R. China; 3 Department of Pathology, Xiangya Hospital of Centre-south University, Changsha, Hunan, P.R. China; 4 Center for Molecular Medicine, Xiangya Hospital of Centre-South University, Changsha, Hunan, P.R. China; Henry Ford Health System, UNITED STATES

## Abstract

Microvascular invasion (MVI) is regarded as one of the independent risk factors for recurrence and poor prognosis of hepatocellular carcinoma (HCC). The presence of MVI in HCCs was evaluated on the basis of pathological reports of surgical specimens and was defined as tumor within a vascular space lined by endothelium that was visible only on microscopy. The aim of the study was to investigate the usefulness of intravoxel incoherent motion (IVIM) diffusion weighted (DW) magnetic resonance (MR) imaging in predicting MVI of HCC. Preoperative IVIM DW imaging and Gd-EOB-DTPA-enhanced MRI (DCE-MRI) of 51 patients were analyzed. Standard apparent diffusion coefficient (ADC), D (the true diffusion coefficient), D* (the pseudodiffusion coefficient) and *f* (the perfusion fraction), relative enhancement (RE) and radiological features were evaluated and analyzed. Univariate analysis revealed that HCCs with MVI had a higher portion of an irregular tumor shape than HCCs without MVI (*p* = 0.009), the Standard ADC, D value were significantly lower in HCCs with MVI (*p* = 0.022, *p* = 0.007, respectively). Multivariate analysis revealed that an irregular shape (*p* = 0.012) and D value ≤ 1.16×10^-3^mm^2^/sec (*p* = 0.048) were independent predictors for MVI. Combining the two factors of an irregular shape and D value, a sensitivity of 94.4% and specificity of 63.6% for predicting MVI was obtained. In conclusion, we found that an irregular shape and D value ≤ 1.16×10^-3^mm^2^/sec may suggest the presence of MVI in HCCs.

## Introduction

Hepatocellular carcinoma (HCC) is one of the most common malignancies and is the third leading cause of cancer-related deaths worldwide [1]. Although the treatment of HCCs is evolving, hepatic resection or liver transplantation (LT) remains the possible treatment to cure HCCs for eligible patients. Nevertheless, the tumor recurrence is 70% after curative resection and 15%-30% after LT at 5 years [2, 3]. Microvascular invasion (MVI), which can be diagnosed only by microscopic observation (mainly in small vessels such as portal vein branches in portal tracts, central veins in noncancerous liver tissue, and venous vessels in the tumor capsule and/or noncapsular fibrous septa)[4], is regard as one of the most well-known independent risk factors for recurrence and poor prognosis [5,6]. Furthermore, the presence of MVI may indicate the necessity of a more extensive resection and neoadjuvant treatments with curative intent [7,8]. Therefore, an accurate preoperative prediction of MVI can help surgeons choose appropriate surgical procedures or select suitable patients for LT based on risk-benefit assessment. However, identification of the MVI requires a definitively histological evaluation of surgical specimens obtained after resection and transplantation, which limits its usefulness on preoperative clinical-decision making [4].

In the past decade, efforts have been made to preoperatively predict MVI of HCC, various potential risk factors have been used like imaging characteristics, serum markers, and clinical predictors. Recent studies even designed a scoring system to predict MVI of HCCs [9–11]. However, there is still great controversy. Recently, several studies have suggested that diffusion-weighted (DW) imaging could be used as a novel technique to predict MVI of HCC [12–14] and drew similar conclusions that HCCs with MVI showed a lower apparent diffusion coefficient (ADC) than HCCs without MVI, although the *b* values used in their studies were different (0,500 sec/mm^2^, 50, 400, 800 sec/mm^2^, 0, 1000 sec/mm^2^; respectively). They all interpreted the reduced ADC values in HCC with MVI with the two plausible reasons: (1) restriction of molecular diffusion and (2) decreased capillary perfusion caused by MVI, although their research failed to definitely separate each other. However, it’s well known that, besides the inaccurate evaluation of ADC value due to the lack of standardization of imaging parameters [15], conventional DW imaging cannot separate and quantify both the molecular diffusivity and the microcapillary perfusion of tissue. Intravoxel incoherent motion (IVIM) DW imaging is a method initially developed by Le Bihan *et al*. [16, 17] to quantitatively assess the microscopic translational motions that occur in each image voxel at MRI, which can distinguish both pure molecular diffusion and microcirculation, or blood perfusion. In the context, IVIM maybe is a more appropriate and accurate technique to preoperatively predict MVI in HCC than ADC, and to testify the aforementioned hypotheses.

Therefore, the aim of our study was to investigate the usefulness of IVIM DW imaging to preoperatively predict MVI of HCC and try to evaluate the probable microperfusion changes caused by MVI.

## Material and methods

### Patients

**Fig 1:**
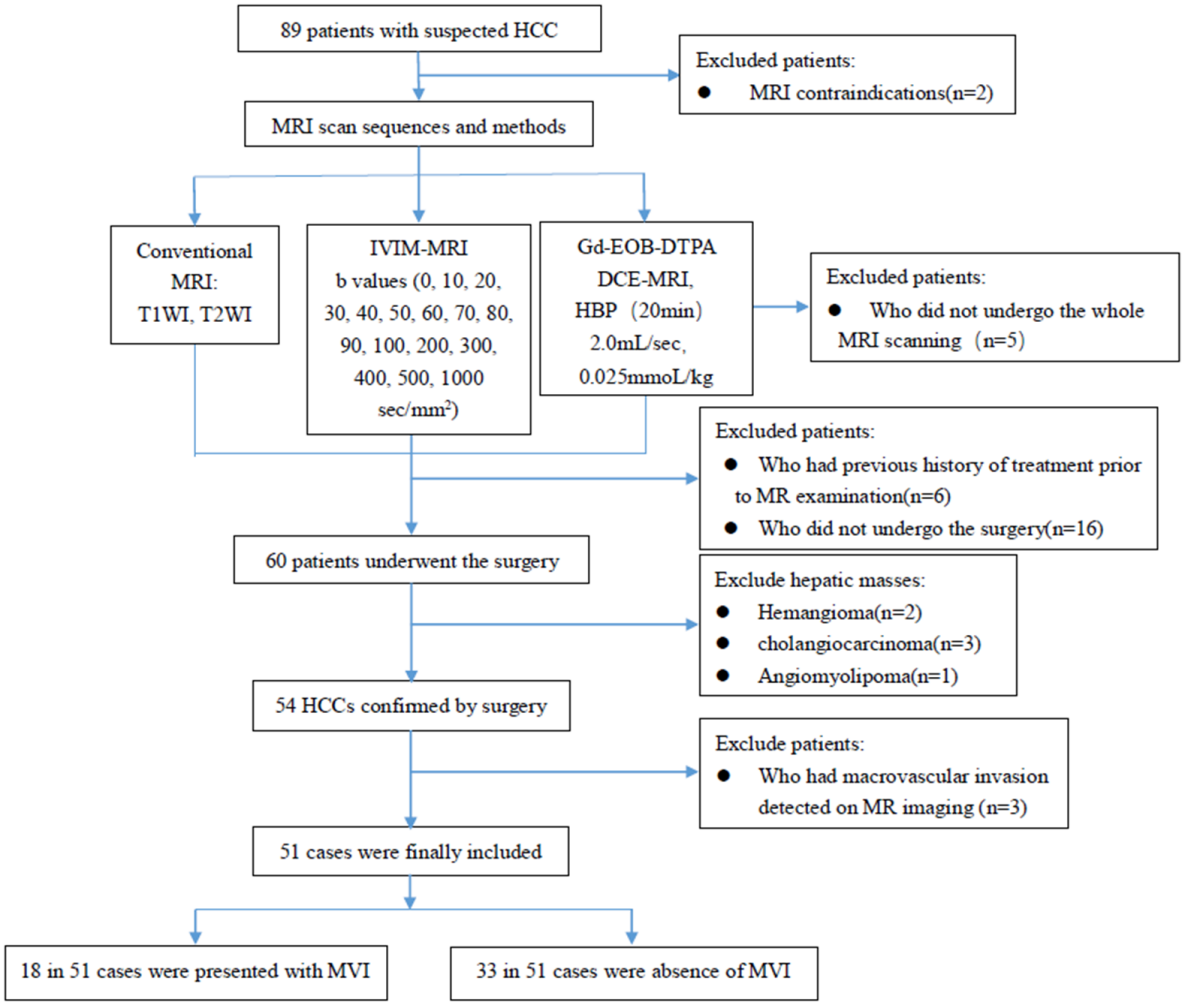
Flowchart of the study population.

Our study was approved by our Medical Ethics Committee of the Xiangya Hospital of Centre South University (IBR NO. 201412452), and the requirement for informed consent was waived because the project was a retrospective study. All methods were carried out in strict accordance with approved guidelines. Between January 2015 and March 2016, 89 patients with suspected HCC were selected for liver MR imaging. Finally, 51 HCCs from 51 patients (mean age ± standard deviation, 50.6 years ± 11.23; range 24–75 years; 43 men and 8 women) were included in our study, according the following inclusion criteria: (1) patients who underwent both IVIM DW imaging and Gd-EOB-DTPA-enhanced MRI (DCE-MRI); (2) a hepatic mass confirmed as HCC by pathological diagnosis after surgery; (3) no treatment for HCC prior to MR examination during the period between MRI examinations and surgery; (4) no macrovascular invasion such as portal vein tumor thrombus was detected on MR imaging and (5) no evidence of extrahepatic metastasis. Of 51 HCCs, 18 were presented with MVI and 33 were absence of MVI (Fig 1). When multiple hepatic masses were present in one patient, we only included the largest tumor with detailed histologic descriptions. The mean time interval between MRI examinations and surgery was 16 days (ranged from 0 to 58 days).

### MR imaging technique

All MRI examinations were performed on GE signa HDx 3.0-T MR (GE healthcare, Grandview, America) with an eight-channel phased array Torso PA coil (GE Healthcare). The routine liver MRI protocols were performed with the following sequences: axial T1-weighted dual-echo in-phase and opposed-phase gradient-echo (GRE) sequence (liver accelerated volume acquisition [LAVA]-Flex; GE Healthcare) and T2-weighted single-shot fast spin echo sequence (SS-FSE) (Table 1). IVIM DW imaging with 16 *b* values (0, 10, 20, 30, 40, 50, 60, 70, 80, 90, 100, 200, 300, 400, 500, 1000 sec/mm^2^) was performed by using a respiratory-triggered single-shot echo-planar sequence (Table 1). Finally, a total of 0.025 mmol/kg of Gd-EOB-DTPA was injected via the antecubital vein at a rate of 2 mL/s with a power injector (Optistar LE; Liebel-Flarisheim Company, OH, Cincinnati, USA) and followed by a 30-ml saline flush. Breath-hold serial axial T1- weighted 3D spoiled GRE fat-suppressed images with slice interpolation was repeated before and seven times after the injection of Gd-EOB-DTPA (Primovist; Bayer HealthCare, Berlin, Germany) (Table 1). In addition to a precontrast acquisition, the dynamic imaging phases including early arterial phase, late arterial phase, early portal phase, late portal phase, the transitional phase and delayed phase (11s/phase, no interval between early arterial phase and late arterial phase, early portal phase and late portal phase), as well as HBP, was performed 10sec, 21sec, 45sec, 56sec, 2min, 5min and 20min after the injection, respectively.

**Table 1:** MR imaging parameters.

Sequence	TR(ms)	TE(ms)	Section Thickness (mm)	Flip Angle (degrees)	FOV(cm)	Matrix	NO. of *b* values	TA	NEX
SSFSE-T2WI	3500	85	6	90	38	288×224	NA	2min57sec	2
IVIM-DW imaging	5714	65.5	6	90	38	96×130	16	5min03sec	1
T1W dual-echo GRE	4.1	2.3/1.1	4	12	38	288×200	NA	14sec	0.7
FS-3D SPGR T1W	2.7	1.3	4	10	38	288×200	NA	11sec/phase	0.72

TR, time of repetition; TE, time of echo; FOV, field of view; TA, time of acquisition; NEX, number of excitations; SSFSE, single-shot fast spin echo; 3D, three dimensional. 16 *b* values: 0, 10, 20, 30, 40, 50, 60, 70, 80, 90, 100, 200, 300, 400, 500, 1000 sec/mm^2^.

### Image analysis

IVIM-derived parameters, relative enhancement (RE) of HBP and all MRI radiological features (the number of tumors, tumor size, and shape, radiologically demonstrated “tumor capsule” appearance, “washout”, intratumoral hemorrhage and intra-lesional fat) were measured and assessed by two radiologists (W.Z.L and X.P.Y, with 12 and 6 years of experience in hepatic MRI, respectively) in consensus. Both observers were aware of the segment location of HCCs according to the pathology report, although they were blinded to the other pathologic characteristics including vascular invasion or the degree of tumor differentiation.

#### IVIM DW imaging analysis

**Fig 2:**
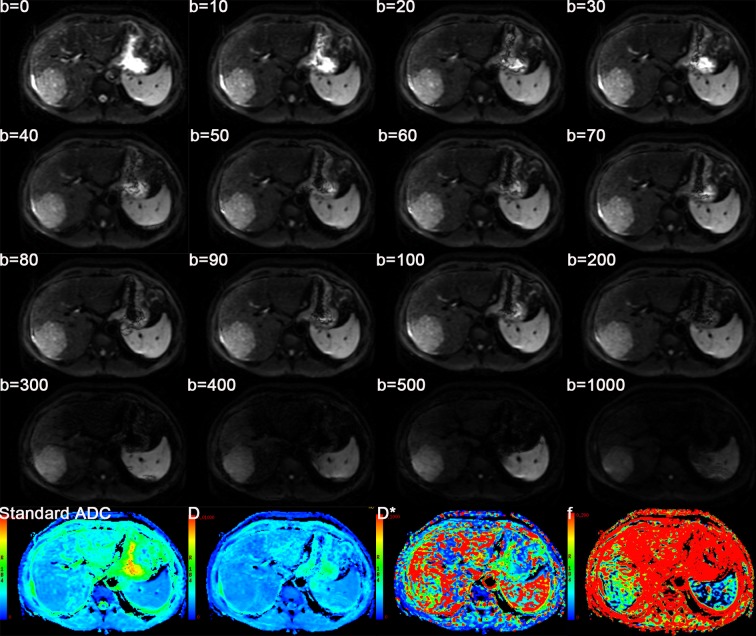
Axial MR image from the 16 b IVIM DWI sequence and IVIM-derived parameters maps of Standard ADC, D, D* and *f*.

Quantitative IVIM-derived parameters, including Standard apparent diffusion coefficient (ADC), D (the true diffusion coefficient), D* (the pseudodiffusion coefficient) and *f* (the perfusion fraction) were automatically extracted from IVIM DW imaging with *b* values of 0, 10, 20, 30, 40, 50, 60, 70, 80, 90, 100, 200, 300, 400, 500, 1000 sec/mm^2^ by using a prototype software program (GE Medical Solutions, MADC, USA) which were fitted on a pixel-by-pixel basis (Fig 2). The technical specifications of IVIM DW imaging parametric map acquisition are explained in S1 Appendix.

#### DCE-MRI analysis

DCE-MRI data were processed with the software on the ADW4.4 workstation (GE, USA). The signal intensity (SI) of the tumor on each phase was measured. The RE between plain SI and contrast-enhanced SI in the HBP was calculated with the following formula [18]: RE = (SI_HBP_−SI_plain_)/SI_plain_.

#### Region of interest (ROI) placement

Circular ROIs were manually positioned by observer 1 (Radiologist X.P.Y) on IVIM parametric maps and DCE-MRI images to measure the quantitative parameters and confirmed by observer 2 (Radiologist W.Z.L). Images of HBP and DW imaging (*b* = 0 sec/mm^2^) were used as references to determine lesion areas on the corresponding DCE-MRI images and IVIM parametric maps. Three ROIs (each approximately 90–130 mm^2^) were placed on a maximum representative slice in the tumor and away from necrosis, cystic, hemorrhage, fat, fiber, blood vessels and bile ducts. For each ROI, the mean value was calculated and values of three ROIs were averaged for each observer’s final measurement. The ROIs drew on IVIM parametric maps and on DCE-MRI images should be as close as possible.

#### Qualitative image analysis

**Fig 3:**
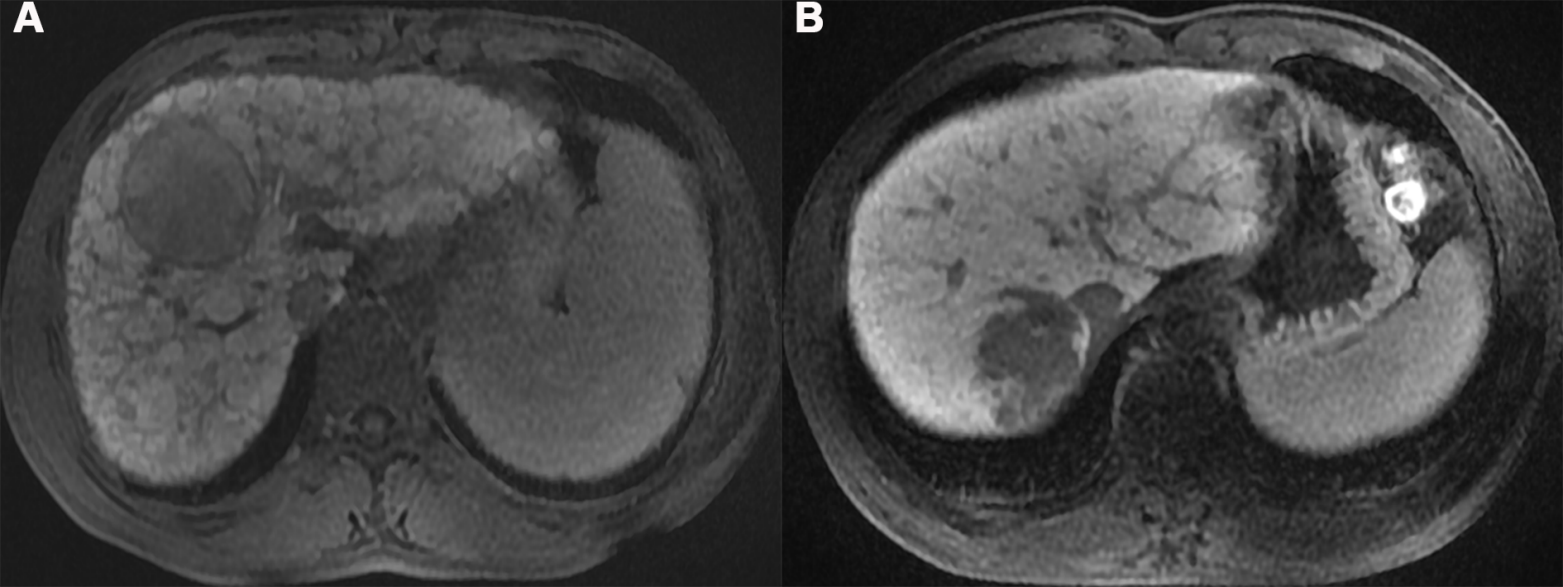
A, a regular shape HCC without microvascular invasion in a 24-year-old man. B, an irregular shape HCC with microvascular invasion in a 52-year-old man.

Tumor shapes were measured on HBP images at all slices and classified as followings[19]: (a) regular shape with a smooth margin, presenting as a round or oval-shaped tumor and intact tumor capsule without extranodular budding into the surrounding liver tissue (Fig 3A) and (b) irregular shape with a no-smooth margin or lobular-shape, presenting as a tumor with an indistinct margin, multi-nodular confluence, or extranodular extension appearance (Fig 3B). The tumor size, defined as the maximum diameter, was measured on HBP images. A rim of increasing enhancement in the portal venous phase or delayed phase around a mass at imaging is termed “tumor capsule” appearance. We categorized the “tumor capsules” into two groups as follows: (a) a present “tumor capsule”, defined as a radiological capsule that completely or partially surrounded the entire circumference of the tumor, and (b) an absent “tumor capsule”, defined as a situation in which no radiological capsule could be identified. “Washout” was visually assessed temporal reduction in enhancement relative to liver from an earlier to a later phase resulting in portal venous phase hypoenhancement or delayed phase hypoenhancement. Intratumoral hemorrhage was assessed by identification of hyperintense on fat-suppressed T1WI. The presence of intra-lesional fat component was assessed with T1-weighted in-phase and opposed-phase chemical-shift imaging. Presence of fat component was demonstrated by a definite decrease of signal intensity between in-phase and opposed-phase images on T1-weighted gradient-echo images. If there was disagreement about the imaging features between the two observers, reevaluation of the imaging features was required to get a final result in consensus.

### Pathology reports

The pathologic reports after surgery were used as a standard of reference for our study. The presence of MVI in HCCs was evaluated on the basis of pathological reports of surgical specimens. Photographs of the main lesion were taken, and the shape, color, texture, segmental location, and size were carefully documented. The following histopathologic factors were assessed for each tumor: gross type, nuclear grade, histologic type, cell type, fibrous capsule formation, vascular (either macrovascular or microvascular) invasion, bile duct invasion, the presence of hemorrhage or necrosis, the presence of satellite nodule and multicentric occurrence. MVI was defined as tumor within a vascular space lined by endothelium that was visible only on microscopy [20]. Histological grade was based on the criteria of the World Health Organization [13]. When the evaluated nodule comprised two areas of different histological grades, the worse histological grade was recorded.

### Statistical analysis

An independent *t* test was used to compare continuous variables, such as the patient’s age, the tumor size, the quantitative parameters from IVIM DW imaging (Standard ADC, D, *f*, and D*) and RE between the present and absent MVI groups. Mann–Whitney *U* test was performed to determine the relationships between serum AFP levels and the presence of MVI. Categorical variables, such as patient sex, background liver parenchymal disease, cause of liver disease, Child-Pugh class, the histological grade, the Barcelona Clinic Liver Cancer (BCLC) stage, the tumor shape, the “washout”, radiologically demonstrated tumor capsule, intratumoral hemorrhage, and intra-lesional fat component were analyzed by using the Fisher’s exact test or the chi-square test. To identify variables associated with MVI of HCCs, logistic regression analysis was conducted. Parameters with a *p*-value of 0.05 or less on univariate analysis were then entered into the multivariate logistic regression analysis in order to elucidate the independent predictors of MVI. Collinearity diagnostics analysis was performed to identify the potential mutilcollinearity between variables. The predictive value of each factor that was significantly different in the multivariate logistic regression analysis for MVI was determined by analysis of the area under the receiver operating characteristic curve(ROC). Odds ratios with 95% confidence intervals for predicting MVI were calculated for each significant factor. A *P* value less than 0.05 was considered significant. MedCalc software (version:11.4.0.2, bvba, Acacialaan, Belgium) was used for ROC analysis, other statistical analyses were performed by using a commercially available software package (SPSS, version 24; SPSS, Chicago, Ill, USA).

## Results

### Demographics and histopathologic results

51 HCCs from 51 patients were enrolled in this study. According to the histopathologic reports, MVI was observed in 18 lesions [MVI (+) group], while 33 lesions had no MVI [MVI (-) group].

With regard to patient age, sex, AFP, tumor size, background liver parenchymal disease, the cause of liver disease, Child-Pugh class, the histological grade or the BCLC stage, there were no significant differences between the two groups. The demographics and histopathologic results were summarized in Table 2.

**Table 2:** Demographic, pathologic and Baseline clinical characteristics.

	MVI (+) (n = 18)	MVI (-) (n = 33)	*P* value
Age (years)^a^	52.00±8.90	49.82±12.42	0.514
AFP(ng/ml)	230.29±315.15	105.27±177.26	0.127
Tumor size(cm)	6.12±3.56	5.43±4.22	0.556
Cirrhosis^b^	11(61.1)	13 (37.5)	0.138
Cause of liver disease			0.397
HBV	10 (55.6)	24 (72.7)	
HCV	3 (16.7)	2 (6.1)	
HAV+HCV	1 (5.5)	0 (0)	
Alcohol	1 (5.5)	3 (9.1)	
Unknown	3 (16.7)	4 (12.1)	
Child-Pugh class			0.65
A	12 (66.7)	24 (72.7)	
B	6 (33.3)	9 (27.3)	
C	0 (0)	0 (0)	
Differentiation			0.064
Well	1 (5.6)	9 (27.3)	
Intermediate	13 (72.2)	22 (66.6)	
Poorly	4 (22.2)	2 (6.1)	
BLCE stage (2010)			0.710
0	0 (0)	1 (3.1)	
A	8 (44.4)	11 (33.3)	
B	10 (55.6)	21 (63.6)	
C, D	0 (0)	0 (0)	

HCC, hepatocellular carcinoma; AFP, alpha-fetoprotein; BCLC, Barcelona Clinic Liver Cancer.

^a^ Data are mean ± standard deviation.

^b^ Data are the number of cases(percentage).

### MRI findings

**Fig 4:**
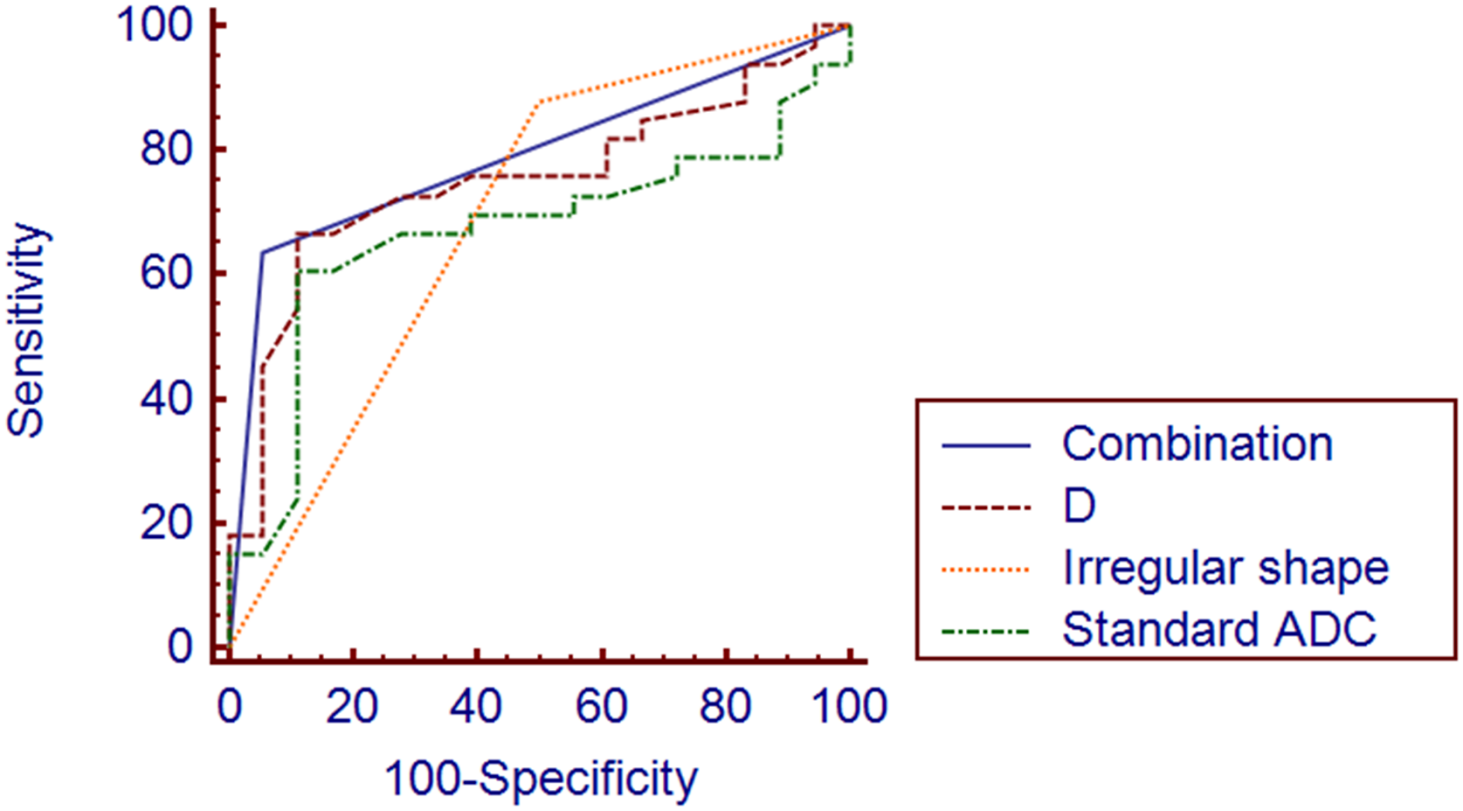
Comparison of ROC curves of irregular shape, Standard ADC, D and the combination of irregular shape and D value for MVI (+) group and MVI (-) group.

**Fig 5:**
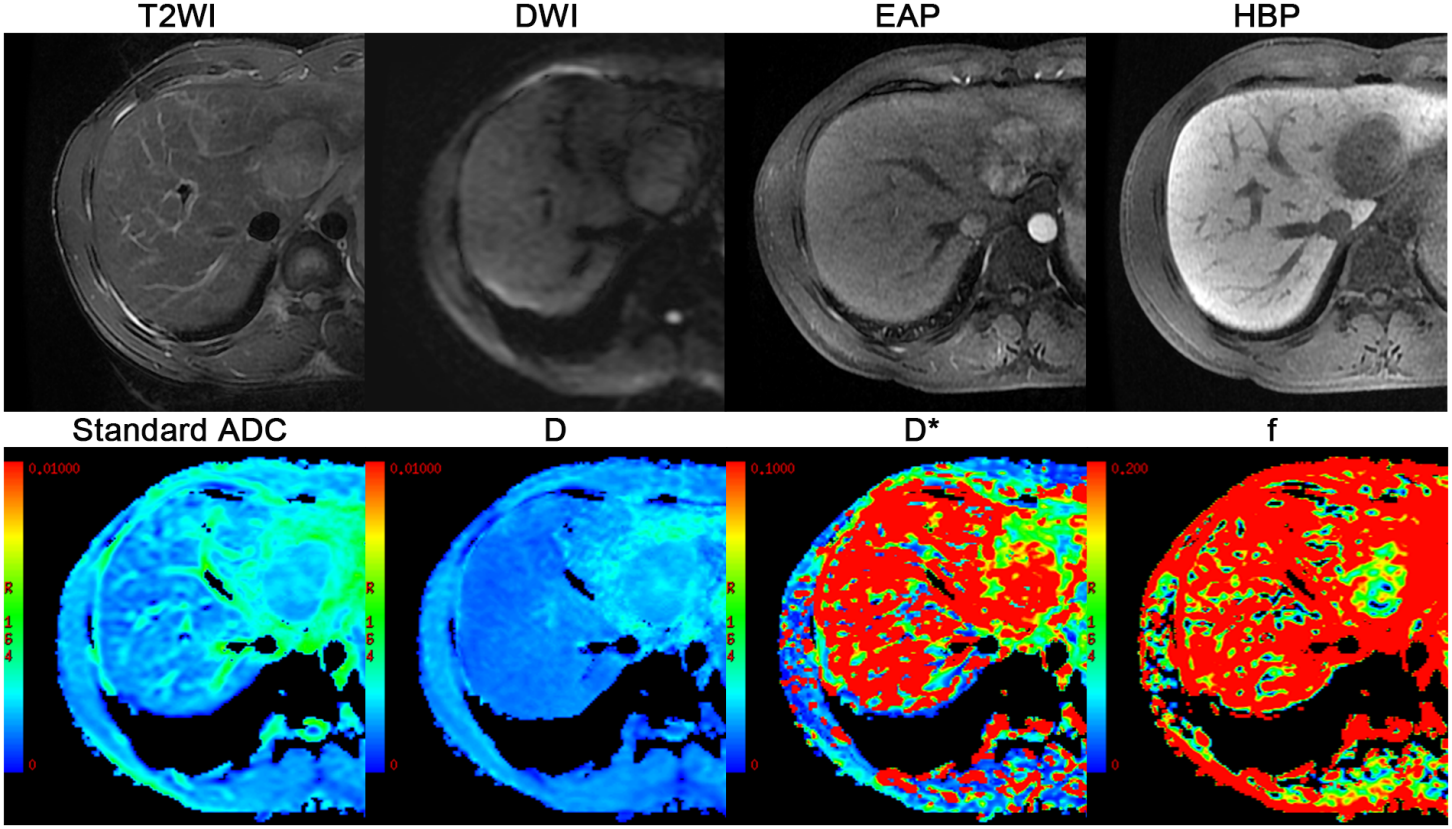
A 28-year-old man with chronic hepatitis B and who underwent liver resection for HCC (moderately differentiation) without microvascular invasion. MRI shows a 4.8 cm hyperintensity on axial respiratory-triggered, T2-weighted, fast-spin-echo (FSE) image (T2WI) in hepatic II with hyperintensity on DW imaging and early arterial-phase(EAP) and a definite hypointensity on hepatobiliary-phase(HBP) images, presented with a regular shape. Corresponding IVIM-derived four parameters (Standard ADC, D, D* *f*) maps, the value of Standard ADC, D, D* f for the lesion was 2.10 × 10^−3^ mm^2^/sec, 1.93× 10^−3^ mm^2^/sec, 83.5× 10^−3^ mm^2^/sec,14.7%, respectively.

**Fig 6:**
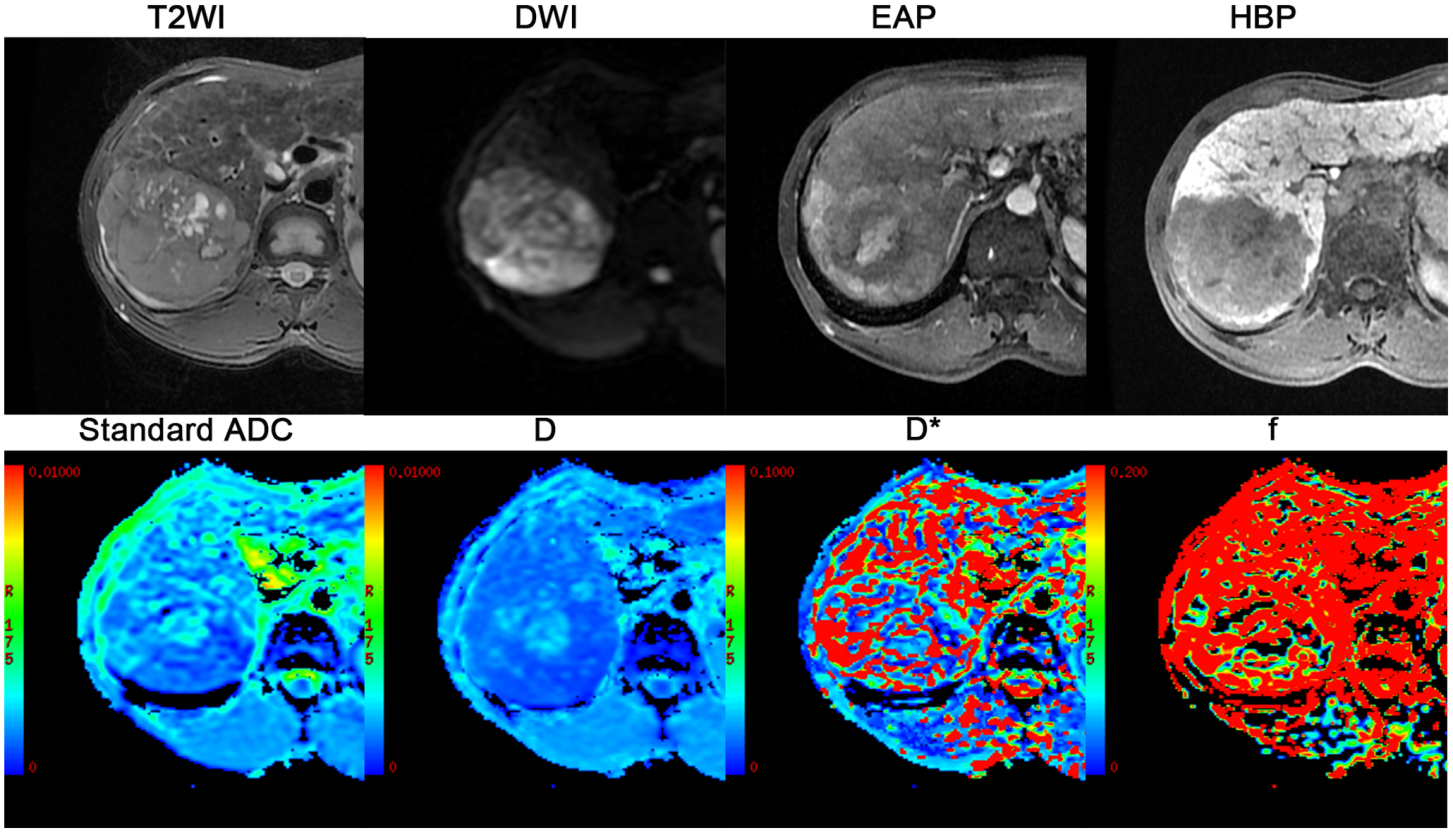
A 45-year-old man with chronic hepatitis B and who underwent liver resection for HCC (poorly differentiation) with microvascular invasion. MRI shows a 9.0 cm hyperintensity on axial respiratory-triggered, T2-weighted, fast-spin-echo (FSE) image (T2WI) in right liver with hyperintensity on DW imaging and early arterial-phase(EAP) and a definite hypointensity on hepatobiliary-phase(HBP) images (d), presented with an irregular shape. Corresponding IVIM-derived four parameters (Standard ADC, D, D* *f*) maps, the value of Standard ADC, D, D* f for the lesion was 1.09 × 10^−3^ mm^2^/sec, 0.89× 10^−3^ mm^2^/sec, 24.3× 10^−3^ mm^2^/sec,12.8%, respectively.

Univariate analysis of the conventional MRI features revealed that the MVI (+) group had a higher portion of an irregular tumor shape (50.0% *vs* 12.1%, *p* = 0.009). In contrast, the presence of radiologically demonstrated tumor capsule, intratumoral hemorrhage, intra-lesional fat, “washout” and multinodular did not show a statistical difference between the two groups (*p* > 0.05). The conventional MRI findings of the MVI (+) and MVI (-) groups were summarized in Table 3. The Standard ADC and D value were significantly lower in the MVI (+) group than in the MVI (-) group (*p* = 0.022, *p* = 0.007, respectively). The D* *f* and RE showed no statistically significant difference between the MVI (+) and MVI (-) group (*p* > 0.05) (Table 4). On ROC analysis, we selected the optimal cut off-value of 1.46×10^-3^mm^2^/s and 1.16×10^-3^mm^2^/s, according to highest Youden index and the areas under the curve (AUC) of Standard ADC and D were 0.670 and 0.753 respectively (Figs 4–6). All parameters of ROC analysis were summarized in Table 5.

**Table 3:** Lesion characteristics and their relationships with MVI.

	MVI (+) (n = 18)	MVI (-) (n = 33)	*P* value
Irregular*	9 (50.0)	4 (12.1)	**0.009**
“Tumor capsule”	8 (44.4)	15 (45.5)	0.945
Intratumoral hemorrhage	4 (22.2)	6 (18.2)	1.000
Intra-lesional fat	2 (11.1)	2 (6.1)	0.923
“Washout”	17 (94.4)	27 (81.8)	0.409
Multinodular	10 (55.6)	11 (33.3)	0.123

Bold indicates *p* < 0.05.

* Data are number of patients, with percentages in parentheses.

**Table 4:** IVIM-parameters, DCE-MRI-parameters and RE with MVI.

	MVI (+) (n = 18)	MVI (-) (n = 33)	*P* value
IVIM-parameters			
Standard ADC	1.35±0.22	1.59±0.49	**0.022**
D	0.99±0.21	1.21±0.29	**0.007**
D*	45.59±21.68	58.99±35.13	0.148
*f*	24.17±13.32	29.67±18.90	0.272
RE	0.56±0.21	0.59±0.23	0.568

Bold indicates *p* < 0.05. Data are mean ± standard deviation.

Unit of StandardADC, D, and D*: 10^-3^mm^2^/sec, unit of f and RE: %.

**Table 5:** Diagnostic performance of all parameters for predicting MVI.

	Cut off	Sensitivity (%)	Specificity (%)	AUC	95%CI
Standard ADC	1.46	60.6	88.9	0.670	0.524–0.795
D	1.16	66.7	88.9	0.753	0.612–0.863

Unit of StandardADC and D: 10^-3^mm^2^/sec. AUC, area under the curve; CI, confidence interval.

### Multivariate analysis

The significant factors (*p* < 0.05) obtained in the univariate analysis were further analyzed to identify independent predictors associated with MVI. Before multivariate analysis performed, we performed the collinearity diagnostics analysis, and no significant multicollinearity was shown between the three variables. Multivariate analysis revealed that an irregular shape (odds ratio = 6.8, 95%CI = 1.53–30.13, *p* = 0.012) and D value of 1.16×10^-3^mm^2^/s or lower (odds ratio = 36.6, 95%CI = 1.03–1292.01, *p* = 0.048) had a statistically significant association with MVI (Table 6). However, both modalities provided insufficient sensitivity and specificity. Specifically, an irregular shape presented on DCE-MRI provided a sensitivity of 50.0% and a specificity of 87.9%, whereas D value ≤ 1.16×10^-3^mm^2^/s on IVIM DW imaging showed a sensitivity of 60.6% and a specificity of 89.9%. D values were changed to categorical variables, that is, more than ≤ 1.16×10^-3^mm^2^/s or less than or equal to ≤ 1.16×10^-3^mm^2^/s. Then we combined irregular shape and D value in parallel, which means that as long as one of the two factors was positive, the final result was considered as positive. After the combination of both imaging modalities, the sensitivity and specificity were improved to 94.4% and of 63.6%, showing a better predictive efficiency for MVI of HCCs (AUC = 0.790) (Fig 4).

**Table 6:** Multivariate analysis of the independent predictive factors for MVI of HCC.

Characteristic	Standard error	Odds ratio	95%CI	*p* value
An irregular shape	0.761	6.8	1.53–30.13	**0.012**
D	1.819	36.6	1.03–1292.01	**0.048**

Bold indicates p < 0.05, CI, confidence interval.

## Discussion

Currently, MVI is an accepted independent predictor of poor survival after hepatic resection and LT [21, 22], with the prevalence ranged from 15–57.1% [23]. It is well known that MVI, known as a histopathological feature, cannot be directly visualized by current imaging technology, which means that accurately preoperative identification of MVI is impossible. However, efforts to preoperatively predict microvascular invasion through investigating the association of imaging findings or clinicopathologic variables with MVI of HCC have not stopped, despite controversy [12, 15, 24–26]. In present study, we have demonstrated that D value and an irregular shape were independent predictive factors for MVI of HCCs.

In the current study, univariate analysis demonstrated that Standard ADC and D were both significantly lower in HCCs with MVI than HCCs without MVI. However, our multiple logistic regression analysis indicated that only D was an independent predictor of MVI, with a sensitivity and specificity of 66.7% and 88.9%, respectively, when the cut-off value of D was 1.16 × 10^−3^ mm^2^/s. It could be explained by that D value may be more accurate to reflect the restriction of molecular diffusion, for that Standard ADC include both the information of molecular diffusion and perfusion. Therefore, the present study verified the hypothesis that the restriction of molecular diffusion is one of the reasons why ADC values reduced in HCCs with MVI. However, it remains difficult to explain why microvascular invasion causes the restriction of molecular. The following mechanism may be a plausible explanation. HCCs with MVI are more likely to differentiate poorly rather than well and moderately differentiated [27], and may have higher cellularity with restricted diffusion than HCCs without MVI. Previous studies also suggested that poorly differentiated HCCs showed lower ADC values than well and moderately differentiated HCCs [28]. However, the histological grade was not a predictor of MVI for HCC in our study, which was identical with several previous studies [12, 14]. The association of ADC value with the histological grade is controversial to date [29]. Theoretically, the histopathologic grade of HCC is determined by both its cellular atypia and its structural atypia. However, according to the theory of DW imaging, only the part of structural atypia can be reflected by ADC value, which could lead the restriction of brownian motion of the extracellular, rather than the intracellular, water molecules. In other words, cellular atypia, mainly determined by the nucleus–cytoplasm ratio, is not fully reflected by DW imaging. Therefore, the decreased molecular restriction in HCC with MVI cannot be interpreted solely by the association between DW imaging and the histological grade, since other factors such as probable architecture changes in the tumor also play a role in restricting the molecular diffusion, and further investigations are needed.

Interestingly, results of IVIM DW imaging suggested that neither the perfusion-related diffusion parameters (D*, *f*) differ significantly between HCCs with and without MVI. Theoretically, it is possible that MVI may cause the decrease of the perfusion by occluding the microvascular. However, liver was a complicated blood supply organ and the perfusion evaluation was determined by the combined effects of hepatic artery, portal vein and hepatic vein. MVI was found in venous vessels usually [4], the perfusion changes caused by MVI was probably limited, as the relatively blood flow velocity was lower in venous vessels than in artery. In addition, subsequently vascular reconstructions after MVI appearance like hepatic artery-portal vein fistula formation and arterialized portal vein formation may also complicate the perfusion changes caused by MVI. Moreover, the presence of MVI may reduce the adhesion of vascular endothelial cells, and then increase the vascular permeability and reduce the portal vein resistance. Instead of reducing the perfusion, these hemodynamic changes may cause increased perfusion. Therefore, the exact hemodynamic changes caused by MVI remains difficult to explain by current image modalities, the aforementioned possible mechanisms required further investigation.

Sumie *et al*. [30] have reported that HCC tumor gross classification was a risk factor for predicting MVI. However, this was only available after resection and LT. Therefore, preoperative radiographic tumor margin was used as a compromise for histological gross classification to predict MVI in clinical research. A simply classification of the preoperative radiographic tumor margins (a smooth margin or a non-smooth margin) was used instead of following the histopathological gross categories in our study, as the challenging of differentiation different histopathologic types based on preoperative images [31]. Our study showed that an irregular shape was another independent predictor for MVI in HCCs, which was consistent with the results of the previous studies [19, 32]. However, Kim et al. have reported the contradictory result [31]. It may be explained by the selection bias.

The introduction of DCE-MRI improves the detection of HCC, especially for the diagnosis of hypovascular HCC that cannot be detected by traditional dynamic studies [33]. Recently, DCE-MRI was also reported to preoperatively predict MVI in HCC. Kim’s study demonstrated that the presence of peritumoral hypointensity seen on the HBP images of DCE-MRI could be useful for predicting MVI with a low sensitivity of 38.3%, although with a high specificity of 93.2% [34]. They explained that peritumoral hypointensity seen during HBP suggested the alteration of hepatic function around tumors with MVI and decreased contrast perfusion caused by tumor thrombus. However, we did not observe the peritumoral hyphointensity in our cases. Okamura *et al*. [13] attempted to use the RE obtained in HBP to predict MVI, however, the presence or absence of MVI was not correlated with RE, as in our study. The HBP of DCE-MRI has been reported to be useful in the evaluation of the tumor macroscopic type [19], which also used as a reference phase to determine the tumor margins in our study.

No other clinical or radiographic features were identified as predictors in significantly predicting MVI. Particularly, tumor size and AFP, which were suggested as predictors for MVI in previous studies [10, 25,26], failed to show a significant correlation with MVI in our study. One possible reason for the discrepancy is the selection bias, with the fact that only surgically eligible cases were included in our study. Furthermore, tumor markers, such as AFP, are highly variable among individuals and might be influenced by the presence of chronic hepatitis C or liver cirrhosis [19]. Therefore, a single tumor mark may not so suitable to predict MVI in HCCs.

Our study had several limitations. Firstly, we were not able to assess MVI on a per-lesion basis in the retrospective design of the study, but rather on a per-patient basis. In addition, we could not correlate the ROIs which we drew on MRI images with pathological vessel invasion on a site-by-site basis. Secondly, the sample size was small and only the cases with hepatic resection were enrolled in our study. Thirdly, the values of parameters obtained from IVIM DW imaging are dependent on the set of *b* values, Therefore, the best cut-off value for predicting MVI may differ between facilities. Thus, it is necessary to standardize the technical protocol to use such cut-off point in the clinic. Future prospective and large sample studies about such techniques with the presence of MVI would be interesting.

In conclusion, preoperative imaging findings such as an irregular shape seen on gadoxetic acid enhanced MR and a D value ≤ 1.16×10^-3^mm^2^/s on IVIM DW imaging could be preoperative surrogate markers for MVI in HCCs and may influence the clinical-decision making to improve the therapeutic outcome and meticulous selecting suitable patients for LT, since the accepted poor resection and post-transplant outcome in HCC with MVI.

## Supporting information

S1 Appendix IVIM DW imaging parametric map acquisition. (DOCX)
