# Supplementary material for: Preoperative prediction of microvascular invasion of hepatocellular carcinoma with IVIM diffusion-weighted MR imaging and Gd-EOB-DTPA-enhanced MR imaging
Source: PLoS One. 2018 May 17;13(5):e0197488. doi: 10.1371/journal.pone.0197488 (PMC5957402; doi:10.1371/journal.pone.0197488)
Supplement: S1 Appendix — (DOCX) [file pone.0197488.s001.docx]

**S1 Appendix. IVIM DW Imaging Parametric Map Acquisition**

The bi-exponential model from an IVIM DW imaging sequence was expressed by the following Equation (1), as described by Le Bihan et al. [1] :

S_b_/S_0_ = (1-*f*) · exp(-bD) + *f* · exp [-b (D + D*)]. (1)

Where S_b_ is the signal intensity in the pixel with diffusion gradient *b*, S_0_ is the signal intensity without diffusion gradient. *f* is the perfusion fraction related to microcirculation, D is the diffusion parameter representing pure molecular diffusion (slow component of diffusion), and D* is the diffusion parameter which represented incoherent microcirculation within the voxel (perfusion-related diffusion, or fast component of diffusion).

Since D* is significantly greater than D [2], its influence on signal decay can be neglected for *b* values greater than 200 sec/mm^2^. In that case, Equation [1] can be simplified and the estimation of D can be obtained by using only *b* values greater than 200 sec/mm^2^, with a simple linear fit Equation (2):

S_b_/S_0_ = exp (-bD). (2)

With the D value determined by using Equation (2), *f* and D* values can be calculated by using a nonlinear regression algorithm based on Equation (1). The Standard ADC, which equals the global ADC mentioned in previous study or the ADC values usually referenced in previous studies [3], was obtained by using only *b* value of 0, 200, 300, 400, 500, 1000 sec/mm^2^ and was fitted to for Equation (3):

Sb/S_0_ = exp (-bADC). (3)

**References:**

1. Le Bihan D, Breton E, Lallemand D, Grenier P, Cabanis E, Laval-Jeantet M MR imaging of intravoxel incoherent motions: application to diffusion and perfusion in neurologic disorders. Radiology.1986; 161: 401-407.

2. Le Bihan D, Turner R, MacFall JR Effects of intravoxel incoherent motions (IVIM) in steady-state free precession (SSFP) imaging: application to molecular diffusion imaging. Magn Reson Med.1989; 10: 324-337.

3. Luciani A, Vignaud A, Cavet M, Nhieu JT, Mallat A, Ruel L, et al. Liver cirrhosis: intravoxel incoherent motion MR imaging--pilot study. Radiology.2008; 249: 891-899.
